# Antimicrobial Composites Based on Methacrylic Acid–Methyl Methacrylate Electrospun Fibers Stabilized with Copper(II)

**DOI:** 10.3390/molecules29122835

**Published:** 2024-06-14

**Authors:** Ana B. da Silva, Suelen P. Facchi, Fabricio M. Bezerra, Manuel J. Lis, Johny P. Monteiro, Elton. G. Bonafé, Adley F. Rubira, Alessandro F. Martins

**Affiliations:** 1 Group of Polymers and Composite Materials, Department of Chemistry, State University of Maringá (UEM), Maringá 87020-900, PR, Brazil; pg55264@uem.br (A.B.d.S.); afrubira@uem.br (A.F.R.); 2 Laboratory of Materials, Macromolecules, and Composites, Federal University of Technology—Paraná (UTFPR), Apucarana 86812-460, PR, Brazil; johnymonteiro@utfpr.edu.br (J.P.M.); eltonbonafe@utfpr.edu.br (E.G.B.); 3 Graduate Program in Agronomy, State University of Maringá (UEM), Maringá 87020-900, PR, Brazil; pg54955@uem.br; 4 Textile Engineering (COENT), Federal University of Technology—Paraná (UTFPR), Apucarana 86812-460, PR, Brazil; fabriciom@utfpr.edu.br; 5 Intexter-UPC, C/Colom, 15, 08222 Terrassa, Barcelona, Spain; manuel-jose.lis@upc.edu; 6 Department of Chemistry & Biotechnology, University of Wisconsin-River Falls (UWRF), River Falls, WI 54022, USA; 7 Department of Chemistry, Pittsburg State University (PSU), Pittsburg, KS 66762, USA

**Keywords:** electrospinning, optimization, Eudragit L100, bactericidal

## Abstract

This study presents fibers based on methacrylic acid–methyl methacrylate (Eudragit L100) as Cu(II) adsorbents, resulting in antimicrobial complexes. Eudragit L100, an anionic copolymer synthesized by radical polymerization, was electrospun in dimethylformamide (DMF) and ethanol (EtOH). The electrospinning process was optimized through a 2^2^-factorial design, with independent variables (copolymer concentration and EtOH/DMF volume ratio) and three repetitions at the central point. The smallest average fiber diameter (259 ± 53 nm) was obtained at 14% *w*/*v* Eudragit L100 and 80/20 EtOH/DMF volume ratio. The fibers were characterized using scanning electron microscopy (SEM), infrared spectroscopy in attenuated total reflectance mode (FTIR-ATR), and differential scanning calorimetry (DSC). The pseudo-second-order mechanism explained the kinetic adsorption toward Cu(II). The fibers exhibited a maximum adsorption capacity (q_e_) of 43.70 mg/g. The DSC analysis confirmed the Cu(II) absorption, indicating complexation between metallic ions and copolymer networks. The complexed fibers showed a lower degree of swelling than the non-complexed fibers. The complexed fibers exhibited bacteriostatic activity against Gram-negative (*Pseudomonas aeruginosa*) and Gram-positive (*Staphylococcus aureus*) bacteria. This study successfully optimized the electrospinning process to produce thin fibers based on Eudragit L100 for potential applications as adsorbents for Cu(II) ions in aqueous media and for controlling bacterial growth.

## 1. Introduction

The rapid industrial and agricultural advancements of recent decades, while beneficial for society, have had detrimental effects on the environment, resulting in water pollution [1]. Water, being a vital resource for sustaining life, is severely impaired by pollutants, posing risks to humans, flora, and fauna. Among the most hazardous contaminants are toxic metals, including copper, cobalt, mercury, lead, cadmium, and nickel [2]. Toxic metals are non-biodegradable and accumulate within the human body, promoting the carcinogenic effect [3]. Copper leads to several kidney and liver damage [4]. However, copper is essential for human health despite its toxicity, playing a critical role in hemoglobin metabolism and enzymatic activities [5]. The Brazilian Health Regulatory Agency (ANVISA, Brasília, Brazil) recommends a daily copper intake for adults of approximately 900 μg.

High levels of copper have been identified in both drinking water and wastewater in Brazil, highlighting the critical need for effective strategies to eliminate this metallic ion from water sources [6]. Several methods exist for the treatment of wastewater contaminated with toxic substances, including chemical precipitation [7], ion exchange [8], membrane filtration [9], reverse osmosis [10], and adsorption [11]. Among these techniques, adsorption is promising due to its potential for cost-effectiveness, high contaminant removal efficiency, and ease of handling of the adsorbent material [12].

Various adsorbents have been studied for removing copper ions from aqueous solutions, including wheat-straw-based adsorbents [4], porous boron nitride [13], chitosan-based films [14], wood fibers enriched with lignin [15], and composite fibers consisting of poly(m-phenylenediamine)/carbon nanotubes prepared by electrospinning [16]. Different methods are available for fiber production, such as casting, forced spinning, biocomponent spinning, and electrospinning. Among these methods, electrospinning has gained considerable attention due to its ease of processing and capacity to produce fibers with small average diameters [17]. Polymeric adsorbent fibers exhibit outstanding properties, such as a high surface-area-to-volume ratio and desirable mechanical properties, making them suitable for a wide range of applications in biomedicine [18], tissue engineering [18,19], environmental remediation [20], filtration [21], and catalysis [22]. 

In this study, adsorbent fibers were developed by electrospinning polymeric solutions containing the cytocompatible copolymer Eudragit L100, which is synthesized through free radical polymerization of acrylic and methacrylic acid and of the corresponding esters [23]. Eudragit L100 is used in the pharmaceutical industry, particularly in tablet coating and as a matrix for sustained drug release systems [24]. It is an anionic copolymer with solubility in aqueous media with a pH higher than 6.0. The copolymer’s chemical structure includes one carboxylic acid side group per repeat unit. Consequently, the adsorption of copper ions (Cu(II)) resulted in complex formation. The complexation enhanced the fiber stability against dissolution by reducing the negative charge density of Eudragit L100 in aqueous media. Eudragit L100 solutions show electrospinnability in ethanol/N,N-dimethylformamide (EtOH/DMF) mixtures. Through optimization of the electrospinning process, thin fibers were successfully produced and applied as adsorbents toward Cu(II) ions in aqueous systems. The resulting fibers were characterized through FTIR, DSC, and SEM. The kinetic mechanism of Cu(II) adsorption was investigated. The complexed fibers exhibited water stability and demonstrated antimicrobial activity against *Pseudomonas aeruginosa* (*P. aeruginosa*) and *Staphylococcus aureus* (*S. aureus*). The presence of copper in the fibers contributed to their bacteriostatic properties. This study presents Eudragit L100 adsorbent fibers with potential antimicrobial activity for the first time, which could be employed to inhibit bacterial proliferation.

## 2. Results and Discussion

### 2.1. Preliminary Electrospinning Results

**Figure 1:**
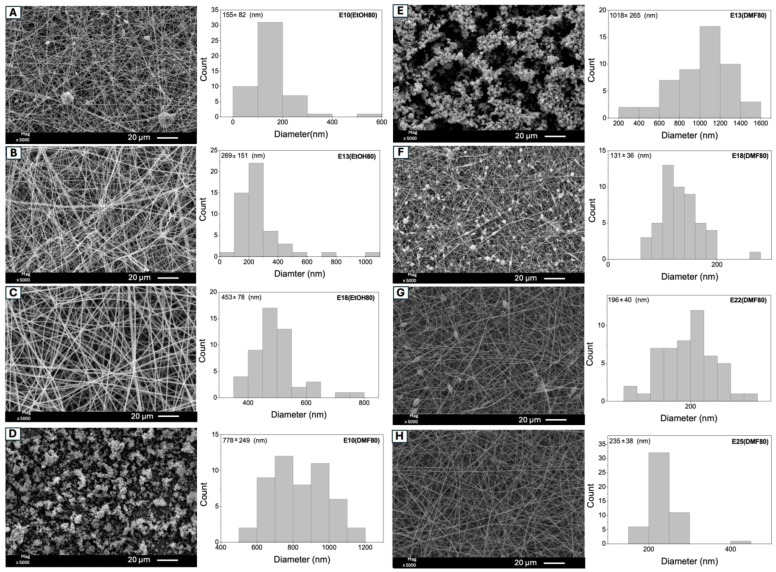
SEM images of Eudragit L100 materials obtained from the experimental conditions compiled in Table 1. The processing parameters used were: applied voltage of 12 kV, flow rate of 0.5 mL/h, grounded static collector (copper plate covered with aluminum foil), syringe with a capillary needle (14 G; 2.1 × 40 mm), and a distance of 10 cm between the needle and the collector. The uppercase letters “A”, “B”, “C”, “D”, “E”, “F”, “G”, and “H” in the SEM images correspond to the samples listed in the first column of Table 1.

Figure 1 displays SEM images of electrospun Eudragit L100 fibers prepared under the conditions outlined in Table 1. The samples are named based on the concentration of Eudragit L100 utilized in the solution preparation, alongside the volume/volume ratio of EtOH/DMF. The letter ‘E’ followed by the numerical value denotes Eudragit L100 and its concentration in solution, while the term within parentheses signifies the solvent in higher concentration in the binary mixture used for solution preparation. For example, sample E10(EtOH80) was derived from a solution of Eudragit L100 at 10% *w*/*v* in a binary EtOH/DMF mixture with 80% EtOH by volume, and sample E13(DMF80) was created from a solution of Eudragit L100 at 13% *w*/*v* in a DMF/EtOH mixture containing 80% DMF by volume. This naming convention extends to the other samples: the letter ‘E’ followed by the numerical value denotes Eudragit L100 and its concentration in solution, while the term within parentheses signifies the solvent in higher concentration in the binary mixture used for solution preparation.

Eudragit L100 solutions at 10, 13, and 18% *w*/*v* were electrospun in an EtOH/DMF mixture at volumetric ratios of 80/20 and 20/80, respectively. When the EtOH content is 80%, the electrospinning of Eudragit L100 solutions at 10 and 13% *w*/*v* results in non-uniform fibers. Fibers produced from the 10% *w*/*v* Eudragit L100 solution exhibit an average diameter of 155 ± 82 nm with beads. In contrast, the 13% *w*/*v* Eudragit L100 solution leads to bead-free fibers; however, their structures are not uniform, supporting an average diameter of 269 ± 151 nm. Conversely, the 18% *w*/*v* Eudragit L100 solution yielded more uniform fibers (453 ± 78 nm) without beads (Figure 1 and Table 1).

With an increase in the concentration from 10% and 13% to 18%, the polymeric chains exhibit a higher degree of entanglement, resulting in larger fiber diameters. As reported in the literature, increasing the copolymer concentration also promotes a higher electrical conductivity. When the polymeric solution has insufficient conductivity, achieving Taylor cone stability becomes more challenging. This affects the stretching of the polymeric jet, as the solution lacks sufficient charge density for fiber stretching, resulting in beaded fibers [25].

When the DMF content is 80% in the Eudragit L100 solution, uniform fibers cannot be obtained within the 10 to 18% *w*/*v* concentration range. Concentrations of 10% and 13% *w*/*v* form beads with mean diameters of 778 ± 249 nm and 1018 ± 265 nm, respectively (Figure 1). In this case, the dominant process is electrospraying due to the low concentration and viscosity of the polymeric solutions. These results indicate that EtOH solvent is more suitable for Eudragit L100 than DMF. 

At an 18% *w*/*v* concentration, the copolymer macromolecules exhibit a higher degree of entanglement, resulting in better dispersion between the solvent and polymeric chains, leading to fibers with an average diameter of 131 ± 37 nm (Figure 1 and Table 1) The degree of polymer chain association is expected to be higher at 18% *w*/*v* in the EtOH/DMF 80/20 mixture compared to the DMF/EtOH 80/20 mixture. Polymer entanglements increase in solution when appropriate solvents are used to prepare the electrospun solution [26,27]. For example, poly(lactic acid) (${\bar{M}}_{w}$ = 26,000 g/mol) solutions in chloroform produced particles via electrospraying at concentrations below 25% *w*/*v*, whereas fibers were formed by electrospinning at 30% *w*/*v* [27]. Therefore, it is proposed to use more concentrated solutions in a DMF/EtOH 80/20 volume mixture to obtain bead-free fibers. Beaded fibers are obtained at a 22% *w*/*v* concentration (mean diameter of 196 ± 40 nm). Conversely, the Eudragit L100 solution at 25% *w*/*v* produces more homogeneous fibers (mean diameter of 235 ± 78 nm) when prepared in the DMF/EtOH 80/20 volume ratio solution (Figure 1 and Table 1). 

The results presented in this study are consistent with those of other studies. For example, Coban et al. conducted a study in which Eudragit L100 fibers were prepared from 20% *w*/*v* concentration in a DMF/methanol 1:9 volume ratio. The fibers were created with a flow rate of 1.0 mL/h, a flat rectangular metallic collector positioned 20 cm away from the needle tip, and a voltage of 15 kV at 24 °C and 20% relative humidity [28]. Despite the different experimental conditions and use of other solvents, the results of Coban et al. align with the present study’s findings. Coban et al. obtained fibers with an average diameter of 620 ± 190 nm from a 20% *w*/*v* Eudragit L100 solution. In this study, fibers with a mean diameter of 453 ± 78 nm were achieved by electrospinning an 18% *w*/*v* Eudragit L100 solution in an 80/20 EtOH/DMF volume mixture.

Giram et al. conducted a study in which Eudragit L100 fibers were prepared from 15% *w*/*v* solutions in an EtOH/DMF volume ratio of 80/20. The electrospinning process was carried out under the following conditions: a distance of 20 cm between the needle tip and the collector, a flow rate of 0.4 mL/h, a voltage of 20 kV, and a temperature range of 25 to 35 °C. These fibers were used as a drug carrier matrix for moxifloxacin hydrochloride for skin repair. Moxifloxacin hydrochloride was added to the polymeric solution at concentrations ranging from 1.5 to 15% *w*/*v*, resulting in fibers with average diameters ranging from 200 to 600 nm [29]. Comparing the results obtained in this study with those of Giram et al., the average fiber diameter increase was observed as the concentration of the polymeric solution increased. The 13% *w*/*v* solution produced fibers with a mean diameter of 269 ± 151 nm, and the 18% *w*/*v* solution resulted in fibers with an average diameter of 453 ± 58 nm. In contrast, Giram et al. achieved fibers with an average diameter of 382 nm using a 15% *w*/*v* Eudragit L100 solution. It is worth noting that these results were obtained using a binary EtOH/DMF mixture solvent.

### 2.2. Fiber Stability

**Figure 2:**
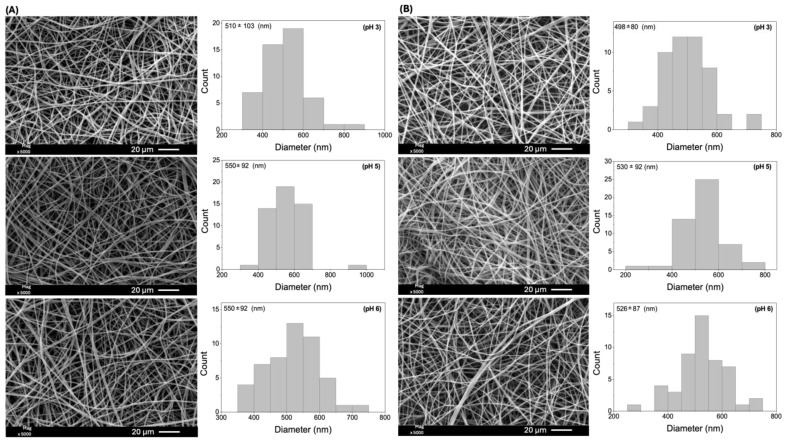
SEM images of E18(EtOH80) fibers (sample corresponding to the uppercase letter “C” in Table 1) after 1 h (**A**) and 5 h (**B**) of immersion in aqueous HNO_3_ solutions at pH 3, 5, and 6.

A fiber stability test was conducted on sample E18(EtOH80), which exhibited an average diameter of 453 ± 78 nm (Figure 1). This sample was selected based on the preliminary electrospinning tests due to its homogeneous and uniform fibers without beads. Moreover, it was obtained from a lower copolymer concentration than sample E25(DMF80) (Figure 1). The stability test involved immersing the fibers in aqueous HNO_3_ solutions with pH values of 3, 5, and 6 for 1 and 5 h. SEM images of the fibers after the stability test are presented in Figure 2.

The SEM images confirm the long-term stability of Eudragit L100 fibers in aqueous media over 5 h. The carboxylic acid groups in the Eudragit L100 repeat units have a pK_a_ value of approximately 6.0. The fibers could exhibit instability at pH 6.0 due to partial ionization of the polymeric chains. However, no signs of dissolution or degradation were observed, indicating the stability of the fibers with a homogeneous and uniform structure. There was some variation in the average fiber diameters compared to the mean diameters determined before the stability test. The average diameter of the as-prepared fiber was 453 ± 78 nm (before the stability test). After the stability test, the diameters increased to 510 ± 103 nm (pH 3), 496 ± 113 nm (pH 5), and 519 ± 78 nm (pH 6). The differences in the average size results were not considered statistically significant (Figure 2).

According to the preliminary results, EtOH/DMF mixtures with a higher proportion of EtOH than DMF provide better conditions for electrospinning Eudragit L100 solutions. Therefore, mixtures with high and adjusted amounts of EtOH were selected to optimize the electrospinning process.

### 2.3. Optimization

As previously mentioned, several parameters play a crucial role in the electrospinning process, including the choice of solvents and the concentration of the polymer [30]. These parameters significantly impact the morphology of the fibers, and it is important to adjust them carefully to prevent beads and achieve thin fibers [31]. When utilizing fibers as adsorbent agents, it is essential to obtain small diameters to maximize the contact surface area between the adsorbate and the adsorbent material [32]. To ensure effective control over this parameter, the statistical method of response surface methodology was employed [31]. Initially, a factorial design (2^2^) was applied, considering two independent variables: Eudragit L100 concentration (14%, 16%, 18% *w*/*v*, denoted as X_1_) and EtOH/DMF solvent ratio (90/10, 85/15, and 80/20 *v*/*v*, denoted as X_2_) (Table 2). 

The model is statistically significant in explaining the data generated from the experimental trials. Analysis of variance confirms the significance of the selected model at a 95% confidence level, with *p*-values below 0.05 and the *F*-test exceeding the tabulated *F*-value (Table 3). Previous studies have also successfully optimized the production of thin fibers using similar parameters to those investigated in this study [33].

The tabulated value for the *F*-test (F_tab_) with three degrees of freedom for the quadratic sum of the regression and the residuals at a 95% confidence level (F_3,3,95_) is 9.28. The experimental *F*-value (F_exp_) obtained from the model is 14.06 (Table 3). Therefore, the model’s significance is supported by the higher F_exp_ value compared to F_tab_. Moreover, the coefficient of determination (R^2^) indicates that the regression model explains approximately 93% of the total variation around the mean. When considering only the contribution of the lack of fit, a parameter directly associated with the model’s efficiency, the model accounts for 98.68% of the presented results. Hence, the model predicts the theoretical outcomes of Eudragit L100 electrospinning, specifically for optimizing the average fiber diameter. 

Equation (1) presents the model regarding coded factors, enabling the fiber diameter (nm) prediction at various levels of each factor. Equation (1) comprises positive terms represented by β_0_ and the interaction factor X_1_X_2_. Negative terms are associated with the pure factors X_1_ and X_2_. These positive and negative terms are related to the influence of factors on the electrospinning process, either increasing or decreasing the fiber diameter. By utilizing Equation (1), the average fiber diameter can be calculated.

(1) $$Fiber\;diameter\left(nm\right)=+498.14-249.25X_{1}-197.25X_{2}+152.25(X_{1}X_{2})$$

The pure factors X_1_ and X_2_ influence the fiber diameter response more than the interaction term X_1_X_2_. The interaction factor does not have the same significance as the pure factors in determining the fiber diameter. This finding is consistent with the statistical analysis presented in Table 3. The experimental *F*-values for factors X_1_ and X_2_ are higher than the tabulated *F*-value, and the probability significance test (*p*) supports values below 0.05, indicating statistical significance at a 95% confidence level. The coefficient of variation for the model was calculated to be 21.79%, which is reasonable given the inherent fluctuations around the mean in diameter measurements at the nanoscale. 

**Figure 3:**
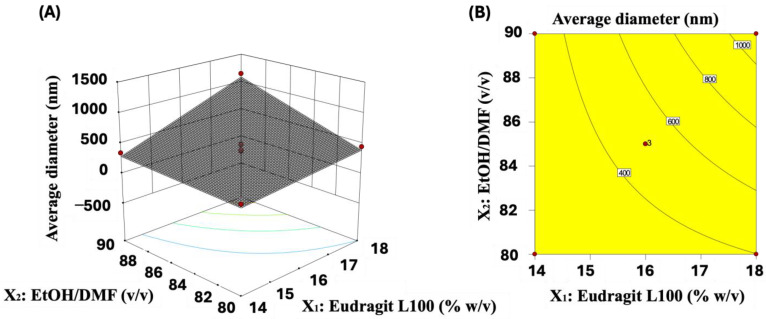
The response surface (**A**) and its corresponding contour plot (**B**) generated using the parameters from Equation (1).

The system’s behavior can be visualized using the response surface and contour plot (Figure 3) through Equation (1). The fiber diameter decreases as the concentration of Eudragit L100 reduces (Figure 3A,B). Higher concentrations of Eudragit L100 lead to more viscous solutions (Table 4), raising the fiber diameters (Figure 3). Increased viscosity is desirable as long as it maintains the stability of Taylor’s cone during the electrospinning process. Solutions with high viscosity prevent solution flow, which can result in needle clogging [27]. Conversely, low viscosity causes Taylor’s cone instability, promoting beaded fibers [34,35]. The response surface demonstrates that thin fibers are produced when the Eudragit L100 concentration is approximately 14% *w*/*v*.

The reduction in the EtOH amount in the EtOH/DMF mixture also correlates with a decrease in fiber diameter. This fact can be attributed to the solution’s conductivity changes as the EtOH/DMF ratio (*v*/*v*) varies from 80/20 to 90/10. The electrical conductivity of the solutions is expected to increase as the amount of EtOH reduces in the EtOH/DMF mixtures, i.e., as the concentration of DMF increases. DMF exhibits superior electrical conductivity compared to EtOH [36]. Consequently, increasing the DMF concentration in the mixture should enhance the electrical conductivity of the solution. This parameter directly influences the formation of thin fibers by facilitating their stretching in the electrospinning process [25]. 

The influence of the variables on the average fiber diameter is depicted in a two-dimensional graph (Figure S1, Supplementary Material). Both variables X_1_ and X_2_ show a similar impact on the response. Decreasing the copolymer concentration and decreasing the amount of EtOH in the Eudragit L100 solution contribute to a reduction in fiber diameter. These trends are observed in Figure S1, where mean fiber diameters ranging from 259 to 1152 nm are observed (Table 2). Assay 1 (X_1_ = 14% *w*/*v* and X_2_ = 80/20 *v*/*v*) yields the thinnest fibers with a diameter of 259 nm, while assay 3 (X_1_ = 18% *w*/*v* and X_2_ = 90/10 *v*/*v*) produces the largest fibers with a mean diameter of 1152 nm (Table 2). These findings align with the previous discussions, emphasizing that decreasing Eudragit L100 concentration and adjusting the EtOH amount are desirable to obtain thinner fibers.

### 2.4. Conductivity and Viscosity 

Viscosity and conductivity measurements were conducted on the Eudragit L100 solutions (conditions investigated in the factorial design, Table 2) and the EtOH/DMF mixtures at ratios of 80/20 and 20/80 (Table 4). The electrical conductivity and viscosity are primarily influenced by the type of solvent, EtOH/DMF ratio, polymer type, and concentration. 

DMF solvent exhibits higher electrical conductivity than EtOH, influencing fiber stretching and diameter. By comparing the experimental conditions of E18(EtOH80) and E18(EtOH90), it is evident that the solution with higher DMF concentration, E18(EtOH80), has the highest electrical conductivity (53.55 mPa·s) and the lowest viscosity (137.25 µS/cm). The increased conductivity results in a higher charge availability on the surface of the polymeric solution, leading to increased repulsion and greater stretching of the polymer jets, resulting in thinner fibers [18]. Similarly, comparing solutions E14(EtOH80) and E18(EtOH80), the viscosity rises with an increasing concentration of Eudragit L100 (Table 4). This viscosity increase occurs due to greater entanglement of the polymeric chains with higher copolymer concentrations, leading to a more viscous solution [37]. 

**Figure 4:**
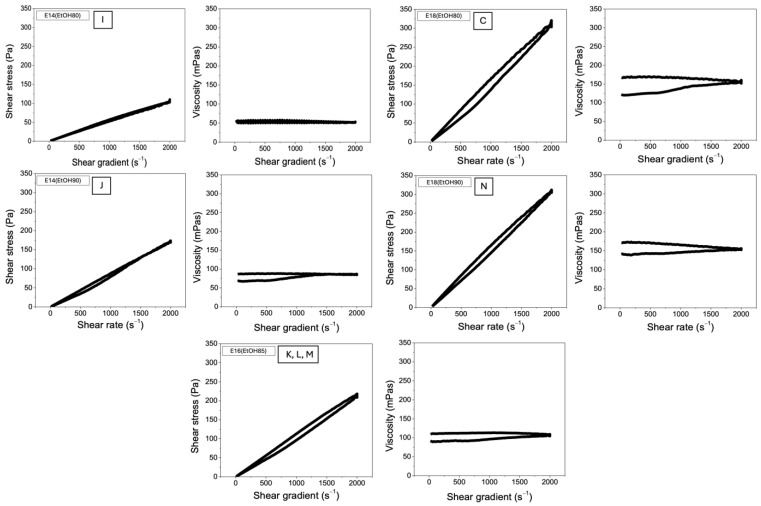
Shear stress and viscosity curves plotted against the shear rate for the polymer solutions used in the optimization study (Table 2). The experimental conditions evaluated include E14(EtOH80), E14(EtOH90), E16(EtOH85), E18(EtOH80), and E18(EtOH90). The uppercase letters (“C”, “I”, “J”, “K”, “L”, “M”, and “N”) following the acronyms in each figure correspond to the samples listed in Table 4.

Figure 4 exhibits the shear stress and viscosity curves for the proposed experimental conditions in the factorial design (Table 2). All the proposed polymeric solutions are classified as Newtonian fluids, whereas the shear stress is directly proportional to the angular deformation rate [38]. Therefore, the shear stress and viscosity curves appear as a straight line. The viscosity remains constant at room temperature and is independent of the applied deformation rate. In contrast, non-Newtonian fluids do not display linearity between shear stress and shear rate. Therefore, their viscosity values can either increase or decrease depending on the specific characteristics of each fluid [38].

**Figure 5:**
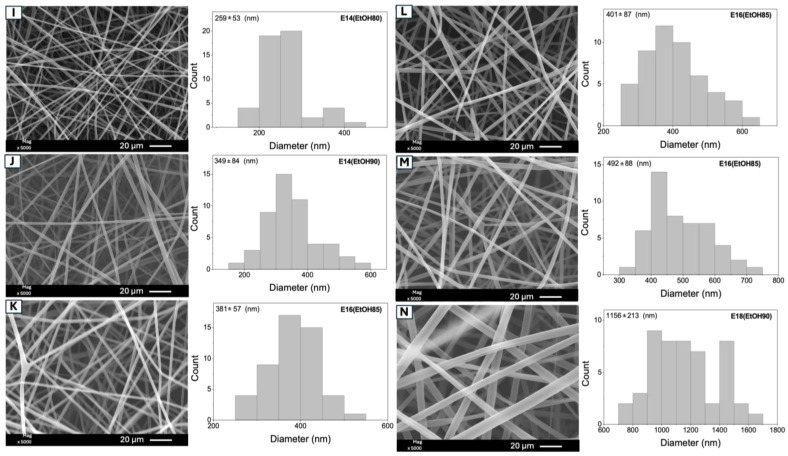
SEM images of the fibers (E14(EtOH80), E14(EtOH90), E16(EtOH85), and E18(EtOH90)) obtained from the experimental conditions investigated in the factorial design (Table 2), excluding sample E18(EtOH80) (label C) previously shown in Figure 1. The uppercase letters “I”, “J”, “K”, “L”, “M”, and “N” in the SEM images correspond to the samples listed in the second column of Table 2.

Figure 5 displays the SEM images of the fibers obtained from the experimental conditions examined in the factorial design (Table 2), excluding sample E18(EtOH80), previously shown in Figure 1. All experimental conditions yielded bead-free fibers. As mentioned earlier, the viscosity and conductivity of the polymeric solutions directly influence the fiber diameter. Among the experimental conditions, sample E14(EtOH80) resulted in fibers with the smallest average diameter (259 ± 53 nm), while solution E18(EtOH90) produced fibers with the largest average diameter (1156 ± 213 nm). The increase in Eudragit L100 concentration from 14 to 18% *w*/*v* led to an approximately 4.5-fold increase in fiber diameter, from 259 nm to 1153 nm. This observation can be attributed to the enhanced entanglement of polymer chains in solution as the copolymer concentration rises, resulting in increased viscosity and higher diameter fibers [39].

Sample E14(EtOH90) exhibits fibers with a larger diameter (349 ± 84 nm) compared to E14(EtOH80) (259 ± 53 nm). This difference is attributed to the EtOH/DMF solvent proportion change. Increasing the DMF concentration enhances the electrical conductivity of the Eudragit L100 solutions, leading to increased stretching of the polymeric jects and, consequently, thinner fibers [40]. A similar effect is observed when comparing the mean diameters of the samples E18(EtOH80) (453 ± 78 nm) and E18(EtOH90) (1156 ± 213 nm). The average diameters of the E16(EtOH85) fibers, which were obtained from the center point experiments, ranged from 381 ± 57 to 482 ± 88 nm.

Other factors, such as environmental parameters (e.g., temperature and relative humidity) may have influenced the variation in these fiber diameters [41]. For example, fibers prepared from a 16% *w*/*v* Eudragit L100 solution at (samples E16(EtOH85 performed in triplicate and denoted by the letters K, L, and M in Table 2) exhibit varying diameters under the same experimental conditions. The average diameters are 381 ± 57 nm (sample K), 401 ± 87 nm (sample L), and 492 ± 88 nm (sample M). Despite these variations, there are no significant differences in average diameter among the samples (Table 2). Such alterations in average diameter are common in fibers obtained by electrospinning and can occur over a wide range. Diameter measurements are taken by selecting random fibers in SEM images, and the results are influenced by many factors, including environmental variables. Even with many controlled parameters, fluctuations in average diameter values are to be expected [35].

### 2.5. Adsorption of Cu(II) Ions 

**Figure 6:**
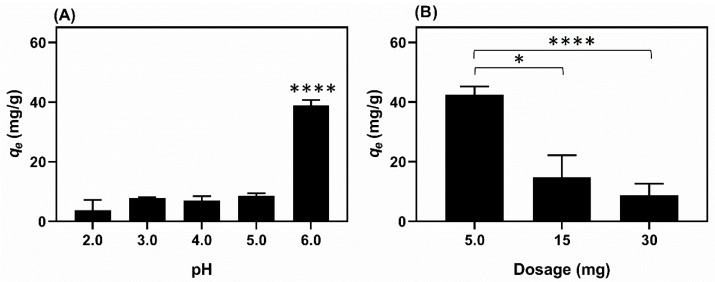
The effect of pH on the adsorption assays is shown in (**A**). The experimental conditions for this investigation included the use of 5 mg of adsorbent (E14(EtOH80), labeled as sample “I” in Table 2), 40 mL of Cu(II) solution (copper(II) sulfate) at 8.0 mg/L, and shaking at 100 rpm (25 °C) for 24 h (**A**). An aqueous HNO_3_ solution (0.01 M) was used to adjust the pH of the solutions in the range of 2.0 to 6.0. The effect of fiber dosage (**B**) on the adsorption of Cu(II) ions in aqueous solutions. The experimental conditions for dosage effect in the Cu(II) adsorption involved the use of 40 mL of Cu(II) solution (copper(II) sulfate) at 8.0 mg/L (pH 6.0) and shaking at 100 rpm (25 °C) (**B**). The terms * and **** indicate results with statistical differences with *p* ≤ 0.05 and *p* ≤ 0.0001, respectively.

Fiber E14(EtOH80) was selected as the adsorbent agent for the adsorption studies due to its higher homogeneity and smaller average diameter compared to the other fibers, which provides a larger contact surface area for adsorption. Figure 6 illustrates the results relating to the influence of pH and dosage on the adsorption process of Cu(II) ions using Eudragit L100 fiber (E14(EtOH80)) as the adsorbent agent. 

The effect of pH on adsorption was investigated using Cu(II) solutions at various pH levels (2.0, 3.0, 4.0, 5.0, and 6.0). pH 6.0 is the most favorable for promoting Cu(II) adsorption from aqueous solutions, achieving a removal efficiency of 37% (q_e_ = 40.2 mg/g). The removal percentage at pH 5.0 is 12.5% (q_e_ = 9.1 mg/g). At pH levels 2.0, 3.0, and 4.0, the removal percentage ranges from 6.8% (q_e_ = 6.2 mg/g) to 8.7% (q_e_ = 8.0 mg/g) (Figure 6A). Investigations at pH values above 6.0 were avoided to prevent the precipitation of copper(II) in the solution [42]. The adsorption of Cu(II) ions is directly influenced by the pH of the solution. Solutions with a pH below 4.0, i.e., more acidic solutions, have protons in excess. This excess reduces the electrostatic interactions between Cu(II) ions and the adsorbent. It also leads to competition between H_3_O^+^ and Cu(II) ions in solution for the polyanion Eudragit L100 chains. As a result, lower pH values correspond to lower removal of Cu(II) ions [43]. 

The dosage effect was investigated by varying the mass of E14(EtOH80) fibers used in the adsorption test. The mass quantities analyzed were 5 mg (0.125 g/L), 15 mg (0.375 g/L), and 30 mg (0.75 g/L) (Figure 6B). The average removal percentages are 56, 72, and 76%, respectively. The removal percentage of Cu(II) increases as the adsorbent concentration rises from 0.125 to 0.75 g/L. Increasing the adsorbent mass raises the number of active sites available for adsorption [44]. For the 5, 15, and 30 mg adsorbent dosages, the average q_e_ values are 42, 15, and 9 mg/g, respectively. Increasing the adsorbent dosage decreases the q_e_ value, which is inversely proportional to the adsorbent mass. Therefore, a dosage of 5 mg (0.125 g/L) was chosen for further adsorption studies. 

**Figure 7:**
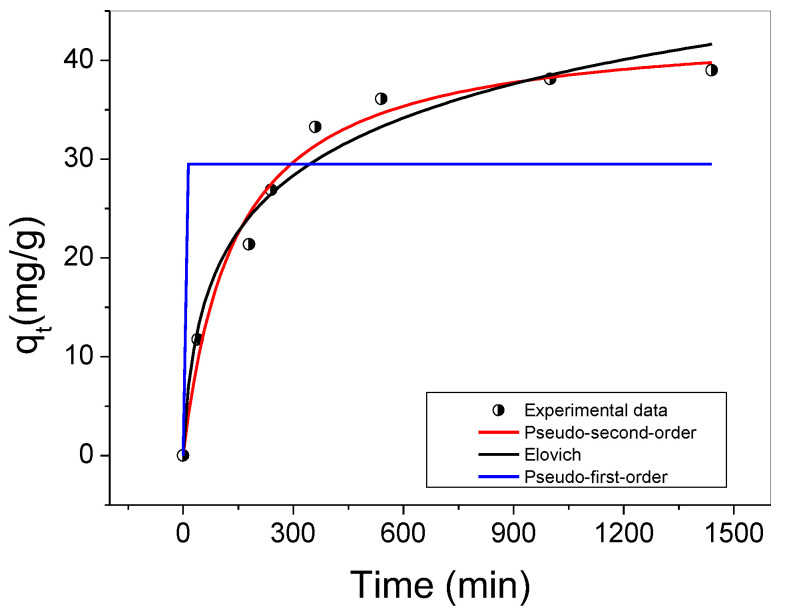
Adsorption kinetics of Cu(II) ions using Eudragit L100 fiber adsorbent (E14(EtOH80)). The figure shows the non-linear fits of the pseudo-first-order, pseudo-second-order, and Elovich kinetic models to the experimental kinetic curve. Experimental conditions: 5 mg of adsorbent (E14(EtOH80), 40 mL of Cu(II) solution (cooper(II) sulfate) at 8.0 mg/L (pH 6.0), 100 rpm, and 25 °C.

The experimental kinetic data for the adsorption of Cu(II) ions is illustrated in Figure 7. Non-linear models such as the pseudo-first order, pseudo-second order, and Elovich models were applied to investigate the adsorption mechanism (Figure 7). The kinetic parameters obtained from the model fitting are summarized in Table 5. The results demonstrate that the pseudo-second-order model best fits the experimental data, with a high determination coefficient (R^2^) of 0.982 and a low ∆q_e_ value of 7.94 (Table 5). The equilibrium state was achieved after approximately 600 min. 

The kinetic adsorption of Cu(II) ions is primarily governed by chemisorption, involving electron exchange or sharing. The carboxylate sites (–COO^−^) in the Eudragit L100 structure can coordinate with Cu(II) ions in aqueous solutions, leading to Cu(II) adsorption [45]. This observation is further supported by the results obtained from FTIR-ATR and DSC analyses. The pseudo-second-order kinetic model, which assumes a constant adsorbate concentration over time, accurately describes the adsorption process. It considers that the total number of binding sites on the adsorbent surface depends on the adsorbate amount adsorbed at equilibrium [46]. This model is commonly associated with adsorption mechanisms that involve multiple steps [47].

The findings of this study are consistent with a previous report by Chen et al. [47], in which polyacrylonitrile fibers containing ethylenediaminetetraacetic acid were electrospun and used for Cu(II) adsorption (with a maximum adsorption capacity of 115.61 mg/g). The pseudo-second-order kinetic model described the adsorption process well [47]. The pseudo-first-order and pseudo-second-order models are widely employed to elucidate the kinetic behavior of copper ion sorption [48].

### 2.6. Fiber and Composite Characterization 

**Figure 8:**
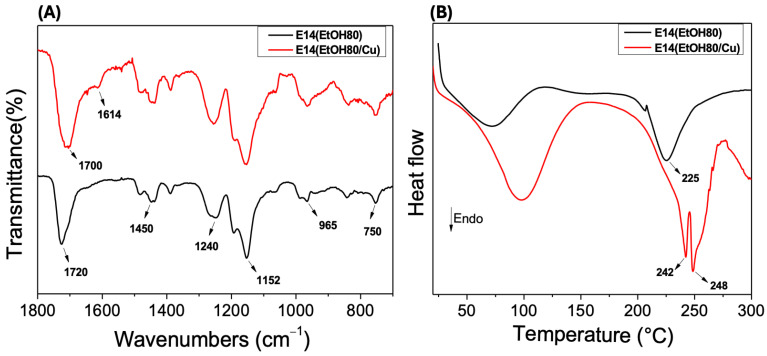
FTIR-ATR spectra (**A**) and DSC curves (**B**) of fibers before (E14(EtOH80)) and after adsorption (E14(EtOH80/Cu)) of Cu(II) ions. Experimental conditions for Cu(II) adsorption were 5 mg of adsorbent, 40 mL of Cu(II) at 8.0 mg/L (pH 6.0), and 100 rpm shaking at 25 °C.

Figure 8A shows the FTIR-ATR spectra of Eudragit L100 fibers before (E14(EtOH80)) and after Cu(II) adsorption (E14(EtOH80/Cu)). The Eudragit L100 fiber FTIR-ATR spectrum exhibits characteristic bands related to the C=O axial stretching of ester groups and carboxylic acids at 1720 cm^−1^. The band at 1240 cm^−1^ is assigned to the C–O axial deformation vibration, whereas the bands at 1450, 965, and 750 cm^−1^ are attributed to C–H angular deformation vibration on the Eudragit L100 chains, respectively.

The E14(EtOH80/Cu) FTIR-ATR spectrum shows characteristic bands of Eudragit L100, similar to those observed in the FTIR-ATR spectrum of the fiber before Cu(II) adsorption. However, a prominent new band at 1614 cm^−1^ occurs in the E14(EtOH80/Cu) FTIR spectrum, which can be attributed to the asymmetric stretching of C=O bonds on carboxylate anions stabilized by metallic ions [49]. This band does not appear in the E14(EtOH80) FTIR-ATR spectrum, suggesting that the Eudragit L100 carboxylate sites interact through Coulomb forces with Cu(II) ions, leading to Cu(II) adsorption [50].

The DSC curves of the E14(EtOH80) and E14(EtOH80/Cu) fibers are presented in Figure 8B. In the E14(EtOH80) DSC curve, the endothermic peak associated with the melting temperature is observed at 225 °C. In contrast, the E14(EtOH80/Cu) DSC profile shows endothermic peaks at 242 and 248 °C. A comparison of the DSC curves reveals distinct profiles for the endothermic peaks, indicating ordered regions in the E14(EtOH80/Cu fiber. Adsorbed Cu(II) ions electrostatically interact with the carboxylate groups of Eudragit L100 within the fibers, leading to Cu(II) adsorption. The intensity of the endothermic peak corresponding to the melting temperature is significantly lower in the E14(EtOH80) DSC profile compared to the intensity of the endothermic peaks in the E14(EtOH80/Cu) DSC curve. The presence of Cu(II) ions results in higher thermal stability of the fibers due to the ordered regions in the complexed fiber, as supported by the endothermic peaks at higher temperatures in the E14(EtOH80/Cu) DSC curve [45] (Figure 8B).

The notable change in the DSC curve profile following Cu(II) adsorption suggests that a portion of the Cu(II) ions are absorbed within the fibers rather than solely adsorbed on the surface. This finding supports the chemisorption mechanism proposed by the kinetic study. In the bulk fibers, each Cu(II) ion can interact with adjacent Eudragit L100 chains, forming a three-dimensional network commonly called “physical hydrogel” [45].

### 2.7. Swelling Degree 

The swelling degree of the fibers E14(EtOH80) and E14(EtOH80/Cu) was determined in an aqueous HNO_3_ solution at pH 6.0 (25 °C) for 24 h. The E14(EtOH80) fiber exhibits a significantly higher swelling degree, reaching 4052 ± 25%, whereas the E14(EtOH80/Cu) fiber shows a much lower swelling degree of only 437 ± 33%. This difference can be attributed to the availability of carboxylate sites in the fiber structure. The E14(EtOH80) samples have carboxylic functions available to interact with water molecules through ion-dipole forces, leading to increased expansion of the polymeric chains and a greater swelling degree. This indicates that the fiber matrix, before adsorption, absorbs an enormous water content.

In contrast, the E14(EtOH80/Cu) fiber is complexed with Cu(II) ions. As a result, the availability of carboxylate groups for interaction with water is reduced. Instead, carboxylate anions in the Eudragit L100 fibers interact through coulombic interactions with Cu(II) ions, resulting in a three-dimensional Eudragit L100 network supported by the physical crosslinking of adjacent copolymer chains with Cu(II) ions. Consequently, the carboxylate ions in the E14(EtOH80/Cu) fiber are no longer accessible to interact with water molecules, significantly reducing swelling. These findings are consistent with the FTIR-ATR and DSC results, supporting the complexation of Cu(II) ions with the Eudragit L100 fibers.

### 2.8. Antimicrobial Activity

**Figure 9:**
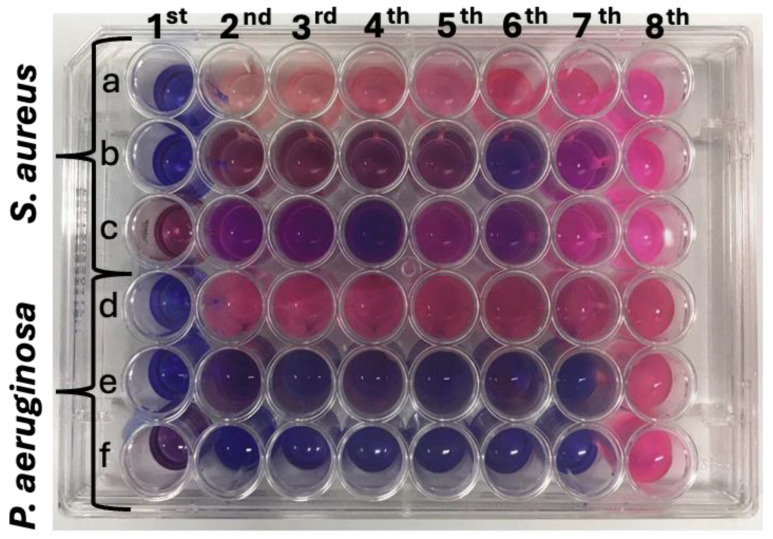
Digital image of a 48-well plate containing microbial cultures in contact with Eudragit L100 fibers (E14(EtOH80) and E14(EtOH80/Cu)), and aqueous copper(II) sulfate solutions. Rows “a”, “b”, and “c” represent experiments performed with *S. aureus*, while rows “d”, “e”, and “f” represent experiments performed with *P. aeruginosa*. Wells in rows “a” and “d” were seeded with E14(EtOH80), wells in rows “b” and “e” were seeded with E14(EtOH80/Cu, and wells in rows “c” and “f” were seeded with aqueous copper(II) sulfate solutions. The first column represents wells of negative control assays containing culture medium and samples without microbial cells. The well in the eighth column indicates the positive control assays corresponding to microbial suspensions without samples (fibers or copper(II) sulfate).

Figure 9 shows a digital image of a 48-well plate containing bacterial suspensions, where E14(EtOH80), E14(EtOH80/Cu), and copper(II) sulfate solutions were added. The first three rows (“a”, “b”, and “c”) correspond to wells seeded with *S. aureus*, while the last three rows (“d”, “e”, and “f”) represent wells seeded with *P. aeruginosa*. The first column represents the positive control, which contains samples without bacteria. The eighth column represents the negative control, consisting of wells containing only microbial culture without samples. Rows “a” and “d” correspond to the assays conducted with E14(ETOH80), while rows “b” and “e” represent the 48-well plates seeded with E14(ETOH80/Cu), and rows “c” and “f” were seeded with copper(II) sulfate (Figure 9). The digital image was captured after adding resazurin, a colorimetric indicator of cell viability. Blue wells indicate low microbial activity, while pink ones indicate high cell viability (Figure 9).

The antimicrobial assays were performed using copper(II) sulfate, E14(EtOH80), and E14(EtOH80/Cu) at similar concentrations (Table 6). Each well in a 48-well plate was supplemented with a predetermined mass of fiber disks or copper(II) sulfate. The concentration of Cu(II) ions within the E14(EtOH80/Cu), which was used in the antimicrobial assay (rows “b” and “e” in Figure 9) is indicated in Table 6. The Cu(II) concentration in the E14(EtOH80/Cu) fibers incubated with the microbial cells was calculated based on the q_e_ value (43.70 mg/g). The E14(EtOH80) fiber does not contain Cu(II) ions in its composition, comprising the fiber before the adsorption of Cu(II) ions. The E14(EtOH80) concentration used in the antimicrobial assay is compiled in Table 6, along with the copper(II) concentration (mg/mL). The concentrations presented in Table 6 are correlated with Figure 9.

The colorimetric assay confirms the lack of cytotoxicity of the E14(EtOH80) fiber against both microbial strains. Wells containing E14(EtOH80) (rows “a” for *S. aureus* and “d” for *P. aeruginosa*) exhibit a pink color, indicating resazurin reduction to resorufin due to increased cellular activity (Figure 9). This confirms the proliferation of microorganisms in the wells with E14(EtOH80) fiber. In contrast, the wells (rows “b” and “e”) seeded with E14(EtOH80/Cu) demonstrate inhibition of microbial growth (Figure 9). The MIC values obtained from the colorimetric assay, resulting in low cell viability, are 9.50 mg/mL (equivalent to 3.32 × 10^−4^ mg/mL of adsorbed Cu(II)) for *S. aureus* and 4.75 mg/mL (equivalent to 1.66 × 10^−4^ mg/mL of adsorbed Cu(II)) for *P. aeruginosa* (Table 6).

The MIC values differ when comparing the concentrations of Cu(II) present in the E14(EtOH80/Cu) fibers and copper(II) sulfate solutions. The MIC values supported by the copper(II) sulfate solution are 2.5 mg/mL for *S. aureus* against 3.32 × 10^−4^ mg/mL of adsorbed Cu(II)) and 1.25 mg/mL for *P. aeruginosa* against 1.66 × 10^−4^ mg/mL of adsorbed Cu(II). It is suggested that these differences primarily depend on the sample type. The E14(EtOH80/Cu) fibers dissolve, releasing Cu(II) ions into the microbial suspension at pH 7.4, while the copper(II) sulfate solution contains sulfate ions that can stabilize the Cu(II) ions, hindering their action against bacteria, as metallic ions have recognized antimicrobial activity in microbial suspension (e.g., silver and zinc ions). Additionally, Cu(II) ions in aqueous media occur in the form of copper(II) complexes, where Cu(II) ions coordinate with six water molecules. This should influence the antimicrobial properties of the samples, justifying the difference in the MIC values.

The results agree with other findings already reported. Chai et al. utilized coated stainless-steel alloys with copper to prevent implant-related infections in bone tissues [51]. Martins et al. [45] developed films of pectin/chitosan through casting and utilized these films as adsorbents for Cu(II) ions (q_e_ = 29.20 mg/g). They demonstrated that the films containing Cu(II) ions exhibited bacteriostatic activity against *Escherichia coli* (*E. coli*) at 25 mg/mL, whereas the film without adsorbed Cu(II) did not show any activity, even at 100 mg/mL. The bacteriostatic activity is dependent on the copper(II) ion in the film, which remained insoluble in the microbial suspension. Deus et al. [52] reported MIC values higher than 2 mg/mL for copper(II) sulfate against many isolates of *E. coli*, a Gram-negative bacterium.

Aliquots were collected from wells that did not show visible growth as indicated by the resazurin (Figure 9) and transferred to Petri dishes (85 × 10 mm) containing Mueller–Hinton agar. The dishes were then incubated at 37 °C for 24 h to determine if the samples exhibited bactericidal activity (Figure S2, Supplementary Material). The bacterial colonies proliferate in the Petri dishes, indicating that the E14(EtOH80/Cu sample and the copper(II) sulfate solution do not possess bactericidal activity against *P. aeruginosa* and *S. aureus* (Figure S3). Therefore, the E14(EtOH80/Cu fiber and Cu(II) solutions exhibit bacteriostatic activity against *P. aeruginosa* and *S. aureus.*

## 3. Experimental

### 3.1. Materials and Methods

Methacrylic acid–methyl methacrylate copolymer Eudragit L100 (125,000 g/mol) was donated by Evonik (Barcelona, Spain). Dimethylformamide (DMF) (99.8%) was purchased from Sigma-Aldrich (São Paulo, Brazil), and absolute ethyl alcohol (EtOH) (99.8%) was acquired from Alphatec (São Paulo, Brazil).

### 3.2. Preliminary Electrospinning Tests and Fiber Stability

The fiber preparation followed the procedure outlined in [29], with some modifications. Eudragit L100 solutions at concentrations of 10, 13, and 18% *w*/*v* were prepared using DMF/EtOH volume ratios of 20/80 and 80/20. The specific experimental conditions employed in the fiber preparation process are detailed in Table 1. The sample designation “E10(DMF80)” represents the electrospun fiber produced from a 10% *w*/*v* Eudragit L100 solution with an 80% DMF volume relative percentage, while “E10(EtOH80)” denotes the fiber obtained from a 10% *w*/*v* Eudragit L100 solution with an 80% EtOH volume relative percentage. The same naming convention was used for the other samples (Table 1).

The electrospinning process was performed at 12 kV and 0.5 mL/h flow rate using an infusion pump (Harvard 2.2.2, Holliston, MA, USA). A static metallic collector comprising a copper plate covered with aluminum foil was employed. The electrospinning solutions were electrospun into a 10 mL syringe connected to a capillary needle (14 G; 2.1 × 40 mm) with a fixed distance (10 cm) between the needle tip and the collector.

### 3.3. Stability Tests

The fiber stability was evaluated by immersing the E18(EtOH80) fiber in aqueous solutions at different pH conditions (pH 3, 5, and 6) for 1 and 5 h. The pH of the solutions was adjusted using an aqueous HNO_3_ solution at 0.01 M. The test did not include pH conditions above 6, whereas the Cu(II) ions interact with –OH^−^ ions to form copper(II) hydroxide precipitate.

### 3.4. Optimizing the Electrospinning Process

A factorial design (2^2^) was applied to optimize the electrospinning process, focusing on the effects of two factors: copolymer concentration (Eudragit L100) ranging from 14% to 18% *w*/*v* (X_1_), and EtOH/DMF volume ratio varying from 4 to 9 (X_2_). The response evaluated was the average fiber diameter (nm). The range of independent variables was determined based on preliminary electrospinning tests. Each independent variable was examined at two levels, with the central point in triplicate. The experimental conditions explored in the factorial design are outlined in Table 2.

The experiments were carried out following the conditions compiled in Table 2, and the obtained results were analyzed using Equation (2)

Y = β_0_ + β_1_X_1_ + β_2_X_2_ + β_12_X_1_X_2_ + ɛ (2)

where the response (Y) represents the average fiber diameter, where X_1_ and X_2_ are the independent variables, β_0_ is the constant coefficient, β_1_ and β_2_ are the linear coefficients, β_12_ is the interaction coefficient between X_1_ and X_2_, and ε the random error associated with the predicted response. The adjusted model efficiency and the significance of the independent variables were evaluated using analysis of variance (ANOVA). The response surface, the model equation, and the *p*-values assigned to each variable were obtained using Design-Expert 7.0 software.

### 3.5. Adsorption Studies of Cu(II) Ions

The optimized fiber E14(EtOH80) was selected as the adsorbent for the adsorption study. A stock solution of Cu(II) (copper(II) sulfate) at 1000 mg/L was prepared, and diluted solutions were obtained from this stock solution. To investigate the effect of pH, 5 mg (0.125 g/L) of the fibers were added to Cu(II) solutions (40 mg/L) at pH 2, 3, 4, 5, and 6. The pH of the aqueous solutions was adjusted with HNO_3_ (0.01 M). The dosage effect was studied using 5 mg (0.125 g/L), 15 mg (0.375 g/L), and 30 mg (0.75 g/L) of the adsorbent fibers in 40 mL of Cu(II) solutions at 8.0 mg/L (pH 6.0). These tests were conducted in polypropylene containers in a shaker at 25 °C with agitation (100 rpm) for 24 h.

The kinetic adsorption assay was performed using 40 mL of Cu(II) solution at 8 mg/L (pH 6.0) over 1440 min. Experimental data were fitted to pseudo-first-order, pseudo-second-order, and Elovich kinetic models (Table S1, Supplementary Material) using non-linear regression analysis in Origin 8.5 software. After the adsorption tests, the remaining solutions were filtered, and the supernatants were analyzed using flame atomic absorption spectroscopy (FAAS), using a Thermo Scientific instrument, model ICE 3000. The maximum adsorption capacity (q_m_, q_t_, and q_e_) at desirable times *t* and equilibrium was determined through Equation (3)

(3) $$q_{m}=q_{t}=q_{e}=\left(\frac{C_{0}-C_{e}}{m}\right)\times V$$

where q_m_, q_t_, and q_e_ represent the maximum amounts of Cu(II) (mg) adsorbed per gram of adsorbent (mg/g), C_0_ is the initial Cu(II) concentration (mg/L), C_e_ represents the Cu(II) concentration at equilibrium (mg/L), V is the volume of the adsorbate solution (L), and *m* represents the fiber mass (g). The adsorption results were evaluated by calculating the determination coefficient (R^2^) and the normalized standard deviation Δq_e_(%), which are defined in Equation (4).

(4) $${\Delta q}_{e}\left(\%\right)=\sqrt{\frac{\sum [(q_{e,exp}-q_{e,cal})/q_{e,exp}]²}{N-1}\times 100}$$

where q_e,exp_ and q_e,cal_ (mg/g) represent the maximum Cu(II) amount adsorbed from the experimental and calculated tests, respectively.

### 3.6. Swelling Degree 

The swelling degree of the fibers (before and after adsorption studies) was evaluated in 50 mL of an aqueous solution at pH 6.0 (optimized condition) for 24 h, under magnetic stirring (50 rpm). The pH was adjusted using a 0.0001 M HNO_3_ solution at 25 °C. After 24 h, the swollen samples were weighed, and their swelling degrees were determined through Equation (5)

(5) $$Swelling\;degree\left(\%\right)=\frac{M_{s}-M_{d}}{M_{d}}\times 100$$

where M_s_ is the mass of the swollen samples and M_d_ is the mass of the dried samples.

### 3.7. Characterization

Viscosity measurements of the copolymer solutions were conducted at 25 °C using a continuous shear rheometer (MARS II Haake, Ober-Moerlan, Germany). Shear analyses were performed with a voltage-controlled rheometer equipped with a parallel steel conical plate geometry of 35 mm and a fixed distance of 0.052 mm. The electrical conductivity of the polymeric solutions was measured at 25 °C using an MS Tecnpon conductivity meter with a cell constant (k) of 1. The conductivity and viscosity of the solutions listed in Table 2 were determined.

The fibers (before adsorption and after the stability test) were characterized through scanning electron microscopy (SEM), a Shimadzu SS 550 instrument at a voltage of 12.5 kV, and a double beam scanning electron microscope (Scios FEI, Brno, Czech Republic). For SEM visualization, the fibers were coated with a thin layer of gold (thickness 10 nm). The fibers (before and after adsorption studies) were characterized through Attenuated Total Reflectance Fourier Transform Infrared Spectroscopy (FTIR-ATR) on a Shimadzu Scientific 8300 instrument (Kyoto, Japan) and differential scanning calorimetry (DSC). The FTIR-ATR analysis covered the range of 500 to 4000 cm^−1^ with 64 scans in a spectrophotometer (Thermo Fisher Scientific Inc, Waltham, MA, USA). The DSC analysis was performed in a Shimadzu DSC60 Plus calorimeter (Kyoto, Japan) at a heating rate of 10 °C/min within a temperature range of 20 to 300 °C, under a 50 mL/min flow rate of argon.

### 3.8. Antimicrobial Assay

The inhibitory concentration (MIC) and minimum bactericidal concentration (MBC) of the optimized fiber (E14(EtOH80)) before and after the adsorption studies, as well as the copper(II) sulfate solution, were evaluated against *S. aureus* (INCQS00015) and *P. aeruginosa* (ATCC27853). The samples were sterilized using ethylene oxide at 40 °C for 2 h. The assay was conducted in 48-well plates containing microbial culture suspensions (400 μL) in Mueller–Hinton medium (Sigma-Aldrich, São Paulo, Brazil) at pH 7.4 and incubated for 24 h at 37 °C. Disks (6.0 mm) of the E14(EtOH80) fibers (before adsorption) and E14(EtOH80/Cu) fibers (after adsorption) were added to the 48-well plate containing suspensions of *S. aureus* and *P. aeruginosa* (400 μL) in Mueller–Hinton agar (Sigma-Aldrich, São Paulo, Brazil). The fiber disks (*n =* 6) had an average mass of 1.9 ± 0.2 mg. Different numbers of fiber disks, including 6, 5, 4, 3, 2, and 1, were added to each well of the 48-well plate. The MIC and MBC of the fibers were evaluated within a concentration range of 2.85 to 4.75 mg/mL. Similarly, the MIC and MBC of the copper(II) sulfate solution were evaluated by adding 200 μL of culture medium and 200 μL of copper(II) solutions in the 1.25 to 40 mg/mL concentration range. After 24 h of incubation, an aqueous resazurin solution (1.0 mg/mL) was added to each well. Resazurin serves as a colorimetric indicator of cellular activity. The blue color of the dye indicates low cellular activity, while pink (indicating the reduced form of the dye) implies high cellular viability and microbial proliferation [45]. The MIC is determined as the lowest concentration, where the resazurin emits blue, indicating low cellular viability. For MBC assessment, 10 μL aliquots from wells with low microbial activity (blue color) were seeded onto Mueller–Hinton agar in Petri dishes (90 × 15 mm) and incubated at 37 °C for 24 h. The MBC is the lowest concentration that prevents colony-forming units (CFU) in the Petri dishes.

### 3.9. Statistical Analysis

The results were statistically analyzed using ANOVA analysis with Tukey’s test, with a significance level of 5% (GraphPad Prism 6.0, Boston, MA, USA).

## 4. Conclusions

This study optimizes the electrospinning process using Eudragit L100 copolymer solutions in binary mixtures of ethanol (EtOH) and N,N-dimethyl formamide (DMF) based on a 2^2^-factorial design. The aim was to obtain thin and uniform fibers without beads. The experimental condition yielding the thinnest fibers (259 ± 53 nm) is identified as E14(EtOH80). This condition consisted of a 14% *w*/*v* Eudragit L100 copolymer solution in an 80/20 *v*/*v* binary mixture of EtOH/DMF. Subsequently, the E14(EtOH80) fiber was used as an adsorbent for Cu(II) ions. The adsorption studies revealed that the adsorption process is governed by chemisorption, and the pseudo-second-order kinetic model best describes the experimental data. Consistent with these findings, the FTIR-ATR spectrum of the fibers after Cu(II) adsorption exhibits a characteristic band at 1614 cm^−1^ assigned to complex formation. The DSC curves also support the complexation between adjacent Eudragit L100 chains and Cu(II) ions, suggesting the formation of a three-dimensional network, a “physical hydrogel” supported by coulombic interactions between Cu(II) ions and carboxylate anions upon adjacent copolymer chain segments. Antimicrobial tests demonstrated that the E14(EtOH80)/Cu fiber (sample obtained after adsorption studies), containing Cu(II) ions, exhibited bacteriostatic activity against *P. aeruginosa* and *S. aureus*, inhibiting their growth without bactericidal effect. Fibers based on Eudragit L100 copolymer can be employed to adsorb Cu(II) ions in aqueous systems and reused as bacteriostatic agents. Potential applications include mask filters and surfaces in hospital environments. This study presents a Cu(II) adsorbent agent with environmental and biomedical significance.

## Figures and Tables

**Table 1:** Experimental conditions for fiber preparation.

	Samples *	Eudragit L100 (% *w*/*v*)	EtOH (mL)	DMF (mL)	Size (nm) **
A	E10(EtOH80)	10	4.0	1.0	155 ± 82 ^a^
B	E13(EtOH80)	13	4.0	1.0	269 ± 151 ^b^
C	E18(EtOH80)	18	4.0	1.0	453 ± 78 ^c^
D	E10(DMF80)	10	1.0	4.0	778 ± 249 ^d^
E	E13(DMF80)	13	1.0	4.0	1018 ± 265 ^e^
F	E18(DMF80)	18	1.0	4.0	131 ± 37 ^a^
G	E22(DMF80)	22	1.0	4.0	196 ± 40 ^d^
H	E25(DMF80)	25	1.0	4.0	235 ± 38 ^b^

* The uppercase letters in the first column correspond to the samples listed in Figure 1. ** Different lowercase letters in the “Size” column indicate results with significant differences (*p* ≤ 0.05).

**Table 2:** The factorial design (2^2^) employed to investigate the influence of independent variables on fiber production.

Assays	Fibers		X_1_ (%) *	X_2_ **	Size (nm)
1	I	E14(EtOH80)	(−) (14)	(−) (80/20)	259 ± 53 ^a^
4	J	E14(EtOH90)	(−) (14)	(+) (90/10)	349 ± 84 ^b^
2	K	E16(EtOH85)	(0) (16)	(0) (85/15)	381 ± 57 ^b,c^
5	L	E16(EtOH85)	(0) (16)	(0) (85/15)	401 ± 87 ^c^
7	M	E16(EtOH85)	(0) (16)	(0) (85/15)	492 ± 88 ^c,d^
6	C	E18(EtOH80)	(+) (18)	(−) (80/20)	453 ± 78 ^d^
3	N	E18(EtOH90)	(+) (18)	(+) (90/10)	1152 ± 213 ^e^

E: Eudragit L100, EtOH: Ethanol; DMF: N,N-dimethyl formamide. Factorial design labels: (−) Lower level corresponding to a concentration of 14% *w*/*v* of Eudragit L100 and an EtOH/DMF volume ratio of 80/20; (0) Central level corresponding to a concentration of 16% *w*/*v* of Eudragit L100 and an EtOH/DMF volume ratio of 85/15; (+) Upper level corresponding to a concentration of 18% *w*/*v* of Eudragit L100 and an EtOH/DMF volume ratio of 90/10. The uppercase letters in the second column correspond to the samples listed in Figure 1 (uppercase letter “C”) and Figure 5 (uppercase letters “I”, “J”, “K”, “L”, “M”, and “N”). * X_1_ = copolymer concentration (% *w*/*v*). ** X_2_ = EtOH/DMF volume ratio. Different lowercase letters in the “Size” column indicate results with significant differences (*p* ≤ 0.05).

**Table 3:** Analysis of variance (ANOVA) for the factorial model.

Source	Sum of Squares	Mean Square	*F*-Value	*p*-Value
Model	4.969 × 10^5^	1.6 × 10^5^	14.06	0.0285
X_1_ (Copolymer concen. (% *w*/*v*))	2.485 × 10^5^	2.485 × 10^5^	21.09	0.0194
X_2_ (EtOH/DMF (% *v*/*v*))	1.556 × 10^5^	1.556 × 10^5^	13.21	0.0359
X_1_X_2_	92,720.25	92,720.25	7.87	0.0676
Residual	35,344.11	11,781.37		
Lack of fit	28,343.44	28,343.44	8.10	0.1045
Pure error	7000.67	3500.33		
Total	5.322 × 10^5^			

**Table 4:** Conductivity and viscosity of the Eudragit L100 solutions and EtOH/DMF mixtures without Eudragit L100.

Samples		Conductivity (µS/cm)	Viscosity (mPa·s)
	EtOH	* 1.4 × 10^−9^	* 0.82
	DMF	* 6.0 × 10^−9^	* 1.08
	EtOH80/DMF20	1.36 **	-
	DMF80/EtOH20	3.16 **	-
I	E14(EtOH80)	47.98	52.83
J	E14(EtOH90)	45.48	86.57
K, L, M ***	E16(EtOH85)	48.08	109.45
C	E18(EtOH80)	53.55	137.25
N	E18(EtOH90)	48.45	153.11

* Conductivity and viscosity according to Smallwood (1996) [36]. ** Measurements performed with the binary mixture EtOH/DMF without Eudragit L100. *** Experiments of the central point: Eudragit L100 at 16% *w*/*v* and EtOH/DMF at 85/15 *v*/*v*. E: Eudragit L100, EtOH: Ethanol; DMF: N,N-dimethyl formamide. The uppercase letters in the firts column correspond to the samples listed in Figure 1 (uppercase letter “C”) and Figure 5 (uppercase letters “I”, “J”, “K”, “L”, “M”, and “N”).

**Table 5:** Kinetic parameters of the pseudo-first-order, pseudo-second-order, and Elovich models obtained from the adjustments shown in Figure 7.

Kinetic Parameters	Pseudo-First-Order	Pseudo-Second-Order	Elovich
q_e_	29.50	43.70	0.735
* k/*α*	k_1_ = 1264.21	k_2_ = 1.62 × 10^−4^	α = 0.116
R^2^	0.48121	0.982	0.969
Δq_e_ (%)	65.72	7.94	9.54

q_e_: Amount (mg/g) of Cu(II) adsorbed in the time interval *t*, before achieving the equilibrium. * Kinetic adsorption constants: k_1_ = min^−1^ for pseudo-first-order model, k_2_ and α = g/mg·mim for pseudo-second-order and Elovich models, respectively.

**Table 6:** The number of E14(EtOH80/Cu) fiber disks (6 mm in diameter) added to rows “b” and “e” in the 48-well plate (Figure 9), the concentration of E14(EtOH80/Cu) fibers (mg/mL) in rows “b” and “e”, and the Cu(II) content (mg and mg/mL) incorporated by adsorption in the E14(EtOH80/Cu) fibers seeded with the microbial suspensions in rows “b” and “e”.

Columns in Figure 9	E14(EtOH80/Cu)				CuSO_4_	E14(EtOH80)
	Fiber Disks	Fibers (mg/mL)	Cu(II) in the Fibers (mg)	Cu(II) Conc. in the Fiber Disks (mg/mL)	(mg/mL)	(mg/mL)
1st *	6	28.5	0.3990	9.97 × 10^−4^	40	28.5
2nd	6	28.5	0.3990	9.97 × 10^−4^	40	28.5
3rd	5	23.75	0.3325	8.31 × 10^−4^	20	23.75
4th	4	19.00	0.2660	6.65 × 10^−4^	10	19.00
5th	3	14.25	0.1995	4.98 × 10^−4^	5	14.25
6th	2	9.50	0.1330	3.32 × 10^−4^	2.5	9.50
7th	1	4.75	0.0665	1.66 × 10^−4^	1.25	4.75
8th *	0	0	0	0	0	0

* “1st” refers to the first column in Figure 9, which represents the negative control assays and indicates wells seeded with fibers (E14(EtOH) or E14(EtOH80/Cu)) at 28.5 mg/L and copper(II) sulfate at 40 mg/mL without microbial cells. Similarly, “8th” refers to the eighth column in Figure 9, which denotes the positive control assay, indicating wells containing microbial suspensions without fibers (E14(EtOH80/Cu) or E14(EtOH80)) and cooper(II) sulfate.

## Data Availability

The data presented in this study are available on request from the corresponding author due to privacy or ethical restrictions.

## Supplementary Materials

The following are available online at https://www.mdpi.com/article/10.3390/molecules29122835/s1, Figure S1: Two-dimensional behavior of variables: concentration of Eudragit^®^ L100 (*X*_1_), and EtOH/DMF ratio (*X*_2_) as a function of fiber diameter; Figure S2: Samples of concentration of E14/Cu nanofibers (9.97 × 10^−4^ mg/mL of copper) *P. aeruginosa* (A) and *S. aureus* (B). Copper solution samples (40 mg/mL *P. aeruginosa* (C) and *S. aureus* (D); Table S1: Kinetic models applied in the adsorption test.
