# Supplementary material for: Antimicrobial Composites Based on Methacrylic Acid–Methyl Methacrylate Electrospun Fibers Stabilized with Copper(II)
Source: Molecules. 2024 Jun 14;29(12):2835. doi: 10.3390/molecules29122835 (PMC11206514; doi:10.3390/molecules29122835)
Supplement: Supplementary file 1 [file molecules-29-02835-s001.zip › molecules-3040004-supplementary.pdf]

Supplementary Material

Table S1. Kinetic models applied in the adsorption test.

Pseudo-first-order	$q_t = q_e[1 - e^{k_1 t}]$
Pseudo-second-order	$q_t = \frac{k_2 q_e^2 t}{1 + k_2 q_e t}$
Elovich	$q_t = \frac{1}{\beta} \ln(1 + \alpha\beta t)$

q_e is the amount in mg·g^{-1} of adsorbate adsorbed in the time interval t, before the equilibrium condition, and at equilibrium
k_1 is the rate constant of the pseudo-first-order kinetic model (min^{-1}).
k_2 is the rate constant of pseudo-second-order kinetics (g·mg^{-1} min^{-1}).
t is the adsorption time (min).
α represents the initial adsorption rate (g·mg^{-1} min^{-1}).
β is the desorption coefficient (mg·g^{-1} min^{-1}).
α and **β** are the Elovich constants.

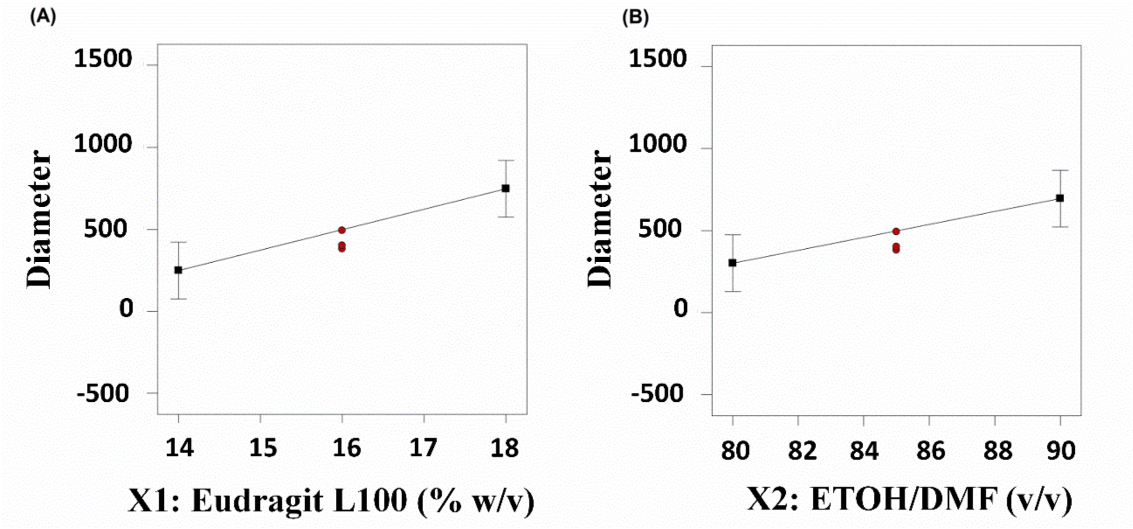

Figure S1. Two-dimensional behavior of variables: concentration of Eudragit® L100 (X_1), and EtOH/DMF ratio (X_2) as a function of fiber diameter.

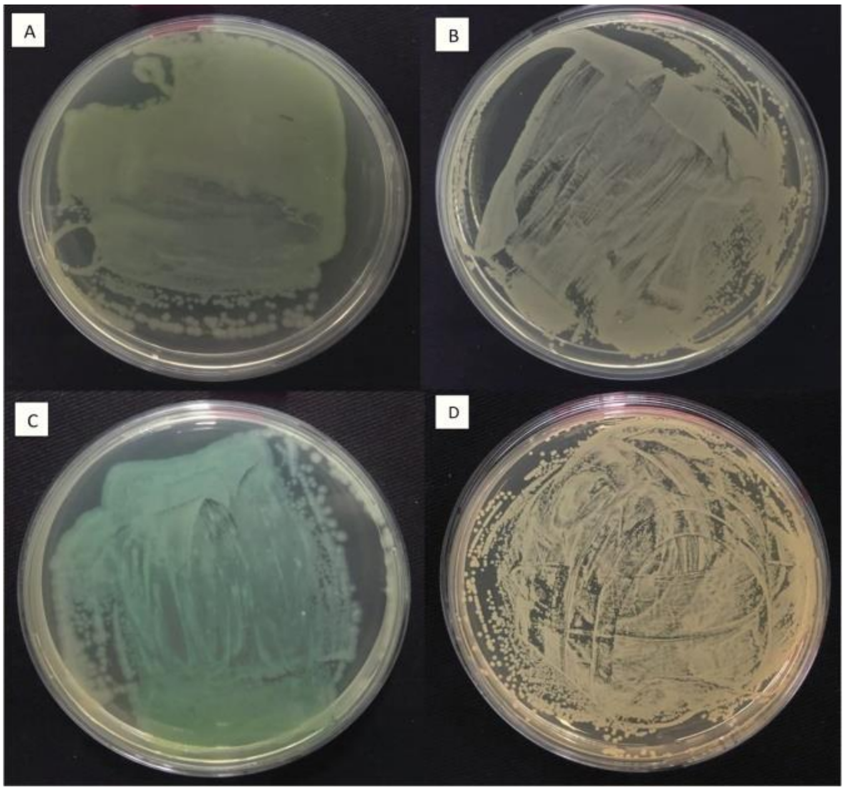

Figure S2. Aliquots (10 μL) of the well seeded with the sample E14(EtOH)/Cu at 28.5 mg/mL were seeded in Petri dishes containing agar. The bacteria *P. aeruginosa* (Figure S2A) and *S. aureus* (Figure S2B) growth on the Petri dishes, confirming that the fibers containing Cu(II) ions obtained after adsorption do not have bactericidal activity. Aliquots (10 μL) of the copper(II) sulfate solution at 40 mg/mL were removed from the wells and seeded in Petri dishes containing agar. The bacteria *P. aeruginosa* (Figure S2C) and *S. aureus* (Figure S2D) growth on the Petri dishes, confirming that the fibers containing Cu(II) ions obtained after adsorption do not have bactericidal activity.
